# The Effect of TiN Coating on the Physicochemical Properties of Ti-13Nb-13Zr Alloy for Biomedical Applications

**DOI:** 10.1021/acs.langmuir.5c00928

**Published:** 2025-05-19

**Authors:** Julia Lisoń-Kubica, Anna Taratuta, Karolina Wilk, Julia Kolasa, Magdalena Antonowicz, Zbigniew Paszenda, Witold Walke, Serap Gümüş, Marcin Basiaga

**Affiliations:** † Faculty of Biomedical Engineering, Department of Biomaterials and Medical Devices Engineering, 49569 Silesian University of Technology, 41-800 Zabrze, Poland; ‡ Department of Metallurgical and Materials Engineering, Kocaeli Üniversitesi, Kabaoǧlu, Baki Komsuoǧlu bulvarı No:515, Umuttepe, 41001 Izmit/Kocaeli, Turkey

## Abstract

Biomaterials used for bone implants should be characterized by good corrosion resistance, appropriate mechanical and electrical properties, high metallurgical quality, biocompatibility, abrasion resistance, and relatively low production costs. Currently used titanium alloys are being replaced by materials with better biocompatibility, so-called new-generation titanium alloys containing elements such as vanadium or niobium. These alloys, with the addition of Zr, Fe, or Ta, give Young’s modulus values approximating that of bone, thus further improving the biocompatibility of the biomaterial. The aim of this study was to assess the impact on its physicochemical and mechanical properties of modifying Ti-13Nb-13Zr alloy with a titanium nitride (TiN) layer using PVD. The knowledge obtained has practical significance for the use of this type of surface modification for various types of miniaturized implants used, for example, in the skeletal system. As part of the assessment of the physicochemical properties of the resulting surface layers, microscopic observations (SEM), potentiodynamic tests, impedance tests, surface wettability tests, scratch tests, and layer abrasion tests were performed. Based on the results obtained, various physicochemical properties of the modified alloy were recorded, depending on the surface modification process. As a result, better properties were noted with respect to corrosion resistance, especially for the tribological properties and the surface resistance to abrasion. The knowledge obtained from this research has practical significance for the use of this type of surface modification for various types of miniaturized implants used in the skeletal system.

## Graphical abstract

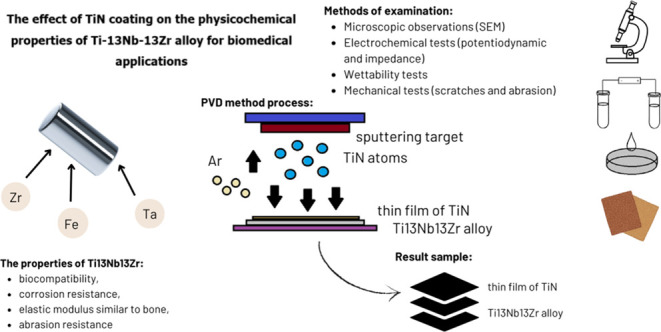

## 1 Introduction

The complexity of the problem related to the use of implants is directly related to their design, the presence or lack of good connectivity, and, above all, the material from which they are made. Both the physical and chemical properties of biomaterials are crucial for assessing how a given material will behave under various conditions, which is particularly important in biomedical applications.^7^ Medical implants are made of a variety of materials, including metals, metal alloys, synthetic polymers, ceramics, hydrogels, and composites.^1^ However, some of the most commonly used materials are titanium and its alloys due to their exhibitive mechanical properties comparable to natural bone.^5^ The Ti-13Nb-13Zr alloy, classified as the β-phase, has a lower β-transus temperature compared to those of the two-phase alloys Ti6Al4 V and Ti6Al7Nb, due to the presence of niobium (Nb) as a β-phase stabilizer. Zirconium (Zr) is a neutral element, not a toxic one, but when combined with Nb, it lowers the value of Young’s modulus to 79 GPa, bringing it closer to that of human bone because of niobium (Nb) β-phase stabilizer properties.^12^ Compared to Ti6Al4 V and Ti6Al7Nb, which have a modulus of 100–110 GPa, Ti-13Nb-13Zr exhibits better corrosion resistance and a lower modulus, making it more suitable for medical applications.^3^

By employing surface modification at the nanotechnology level, scientists have the opportunity to improve, among others, the Ti-13Nb-13Zr alloy by influencing its physicochemical parameters with the use of TiN layers; this improvement has been known for over 30 years.^4^ Surface modification with this layer improves corrosion resistance and reduces the coefficient of friction and the hardness of the base material.^2,4^ One of the most effective ways to ensure a uniform layer distribution on biomaterials, including Ti-13Nb-13Zr, is physical vapor deposition (PVD). This technique allows precise control of the thickness and morphology of the layer, which contributes to an even and homogeneous protective layer. The use of the PVD method in the surface modification of Ti-13Nb-13Zr allows for a significant improvement in its tribological and corrosive properties, which increases the durability and effectiveness of this material in medical applications.^4,7,9,10^ Szymkiewicz and his team performed a modification of the Ti6Al7Nb alloy, focusing on the comparison of the microstructure of the TiN layer.^8^ The PVD method was used by Tillmann et al. to modify additively produced Ti6Al7Nb with alumina (Al_2_O_3_).^10^

Due to the lack of information about the new-generation alloy, we undertook to work on this material, depositing oxide layers on the surface using the ALD (Atomic Layer Deposition) method, by which matter can be coated layer by layer on the substrate surface in the form of a single atomic film. This process precisely controls the deposition of single atoms and nanoclusters, improving the alloy’s tribological and functional properties, which is important for orthopedic implants.^6^ Despite numerous studies on surface modifications of titanium alloys using various techniques, there are no scientific papers focusing on the modification of Ti-13Nb-13Zr with a TiN layer by using the PVD method. The employment of this technique in the application of TiN layers to a next-generation titanium alloy represents a novel approach that has not previously been widely studied or described in the literature. The development of this type of modification is aimed at obtaining significant benefits, especially in the context of improving the tribological and corrosive properties and also the biocompatibility of materials used in medical applications.

## 2 Materials and Methods

In this study, specimens of the Ti alloy (Ti-13Nb-13Zr) were sourced from a bar with a diameter of 14 mm. The chemical composition of the alloy is presented in Table 1. The specimens were ground with progressively finer grades of sandpaper (500, 1200, and 2000 grit) and then electropolished using a phosphate sulfate solution. Following this, a TiN layer was applied to the polished specimens using PVD, with argon being employed to create the plasma, introduced at a power rate of 25%. To evaluate the effectiveness of this surface modification technique, we conducted a series of examinations.

**Table 1:** Chemical Composition of Ti-13Nb-13Zr (Based on the Manufacturer’s Certificate)

C	Fe	N	O	Zr	Nb	H	S	Ti
Percentage by Weight (%)								
0.035	0.085	0.019	0.078	13.49	13.18	0.055	<0.001	73.06

### 2.1 Potentiodynamic Method

In the initial phase, tests for pitting corrosion resistance were conducted using the potentiodynamic method using an AUTOLAB PGSTAT302N potentiostat with Nova 2.1 software. The reference electrode used was Ag/AgCl with a 3 M potassium chloride (KCl) solution, while a platinum rod served as the auxiliary electrode. The scan rate was 3 mV/s. These tests facilitated the examination of the structural characteristics of the layers, including potential defects, sealing issues, substrate reactivity, and the existence of barrier properties against the electrolyte. The resistance to pitting corrosion was evaluated in a saline solution (phosphate-buffered saline, PBS) to simulate the natural tribological conditions found in the human body (Table 2). In the first stage of the study, the open circuit potential (*E*_OCP_) was measured. After the potential stabilized, recording of the curves began potentiodynamically, proceeding in three stages: Registration of the curve from the potential *E*_start_ = *E*_OCP_ to the potential *E*_stop_ = *E*_OCP_ + 200 mV, with a scan rate of 0.1 mV/s. Based on the recorded curve Stern method, the corrosion potential *E*_kor_ was determined.

**Table 2:** PBS Solution Composition

component	concentration [mol/dm^3^]
NaCl	0.14
KCl	0.0027
phosphate ions (PO_4_^3–^)	0.01

### 2.2 Electrochemical Impedance Spectroscopy (EIS)

To gather further insights into the physicochemical properties of the surfaces of the analyzed specimens, an EIS was conducted. Measurements were performed using an AutoLab measurement system, which included a 302N PGSTAT FRA2 (Frequency Response Analyzer) module. This system allowed for experiments across a frequency range of 10^–3^ to 10^4^ Hz. The impedance spectra of the specimens were presented as Nyquist diagrams for various frequency values as well as Bode diagrams. The EIS spectra were analyzed using the least-squares method to fit them to an equivalent electrical circuit. Based on this analysis, the resistance (*R*) and capacitance (*C*) of the systems were determined.

### 2.3 Scratch Test

The scratch test involved creating a scratch using a Rockwell diamond cone as the penetrator with the normal force being gradually increased. The tests were conducted with a loading force ranging from 0.03 to 30 N, utilizing the following parameters: a loading speed of 10 N/min, a table speed of 1 mm/min, and an approximate scratch length of 3 mm.

### 2.4 Tribology

Tribological wear tests were performed to analyze the friction, wear, and surface adhesion. The abrasion tests utilized a tribometer, applying a force load of 5 N, which obtained specific values for the abrasion coefficient. These tests were conducted based on the technical parameters below. Al_2_O_3_ balls with a diameter of 6 mm served as counter specimens in the friction pairs during the tests. The parameters of all tests are listed in Table 3.

**Table 3:** Parameters of Tribological Test

parameter	friction pair
	Al_2_O_3_ ball-disc Ti-13Nb-13Zr
load [N]	5
linear speed [cm/s]	2.83
cycle [-]	1000
frequency [Hz]	80

### 2.5 Scanning Electron Microscopy Imaging

Microscopic observations after tribological testing were conducted using scanning electron microscopy (SEM, Tescan VEGA). The tests were performed under high vacuum conditions at a voltage of 15 kV, utilizing a backscattered electron (BSE) detector. Images captured at various magnifications allowed for the assessment of debris traces formed during the wear test.

### 2.6 Wettability

To assess the wettability of the specimen surfaces, the contact angle method was conducted on selected specimens using the sitting drop technique. Measurements of the contact angle were conducted with 1.5 mm^3^ of distilled water, using a Surftens Universal optical goniometer (OEG). Surftens 4.5 software was used to analyze the captured drop images. Measurements were taken at room temperature (23 ± 1 °C) over a period of 60 s, with a sampling rate of 1 Hz. The data obtained revealed the varying physicochemical properties of the antibacterial films formed during surface modification.

## 3 Results and Discussion

### 3.1 Potentiodynamic Method

Following surface preparation (grinding and polishing) and application of the TiN layer using PVD, the specimens underwent potentiodynamic tests to evaluate their resistance to pitting corrosion in PBS. The results of these tests are presented as polarization curves (Figure 1). The tests conducted provided insights into the impact of surface treatment on corrosion resistance. Furthermore, the surface modification affected the measurement parameters with the corrosion resistance values of the tested specimens being linked to their corrosion potential (*E*_corr_) and the current density.

**Figure 1:**
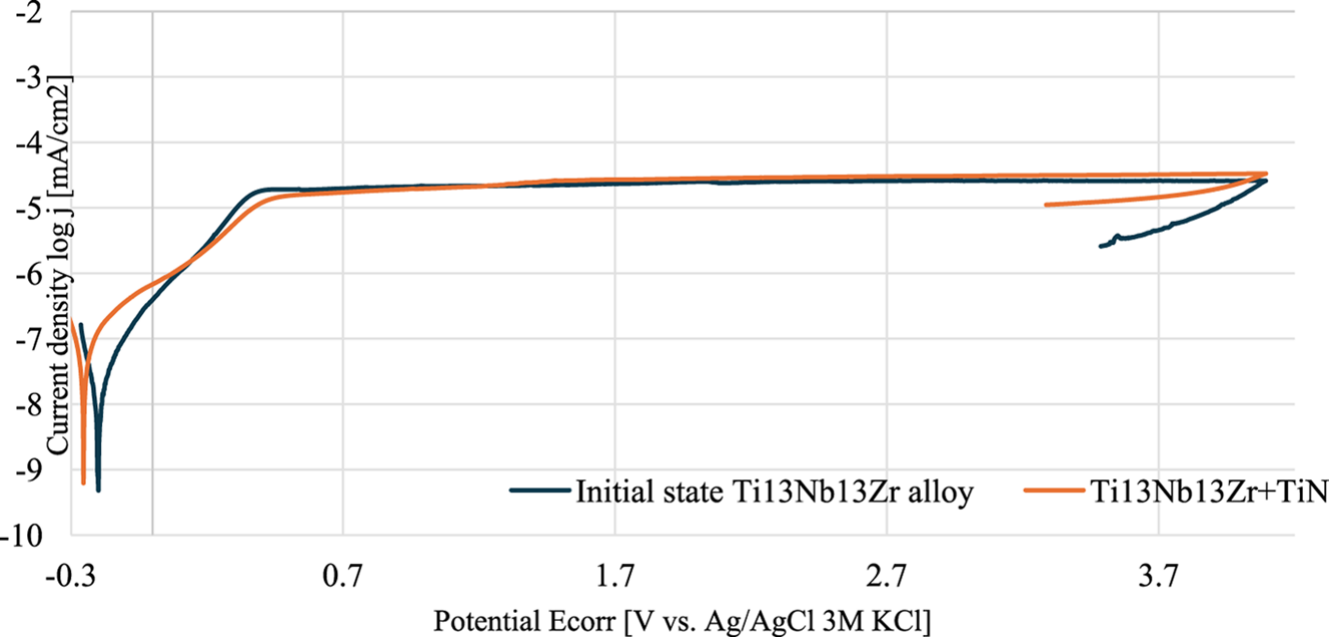
Potentiodynamic curves of the initial state of the Ti-13Nb-13Zr and Ti-13Nb-13Zr + TiN. Polarization curves showing the comparison of corrosion resistance between the untreated and TiN-coated Ti-13Nb-13Zr alloy.

Table 4 summarizes the values for the corrosion potential (*E*_corr_) and current density. Analyzing the polarization curves showed that the Ti-13Nb-13Zr specimen with a TiN layer exhibited slightly higher polarization resistance (*R*_p_) values, indicating improved corrosion resistance compared to the untreated Ti-13Nb-13Zr specimen. Considering the *E*_corr_ parameters, Ti-13Nb-13Zr + TiN gave a similar value (see Table 5).

**Table 4:** Potentiodynamic Curves of the Initial State of Ti-13Nb-13Zr and the Alloy with a TiN Layer

	polarization data	
specimen	*E*_corr_ [mV]	*R*_p_ [kΩ·cm^2^]
Ti-13Nb-13Zr	–200	1.15
Ti-13Nb-13Zr + TiN	–259	1.35

**Table 5:** EIS Examination Results

			CPE_pore_			CPE_dl_	
specimen	*R*_s_, [Ω·cm^2^]	*R*_pore_, [Ω·cm^2^]	*Y*_0_, [Ω^–1^ cm^–2^ s^–*n*^]	*n*	*R*_ct_, [kΩ·cm^2^]	*Y*_0_, [Ω^–1^ cm^–2^ s^–*n*^]	*N*
Ti-13Nb-13Zr	87		0.7968	0.90	347	0.2195	0.93
Ti-13Nb-13Zr + TiN	13	831	0.1441	0.82	314	0.1840	0.94

### 3.2 Electrochemical Impedance Spectroscopy (EIS)

To determine the characteristics of the TiN layer formed on the surface of the Ti-13Nb-13Zr specimens, an EIS was conducted. Typical impedance spectra for the Ti-13Nb-13Zr + TiN specimens prepared via PVD are shown in Figure 2. The electrical properties derived from these spectra are summarized in Table 5. Both Nyquist diagrams revealed distorted semicircular fragments near the origin of the coordinate system, which sometimes transitioned into a linear relationship between the imaginary (*Z*″) and real (*Z*′) components of the impedance (Figure 2, impedance spectra for the Ti-13Nb-13Zr alloy with TiN layer (a) Nyquist and (b) Bode diagrams). The tests indicated that the impedance modulus of the systems decreased with increasing frequency regardless of the substrate type and TiN layer parameters.

**Figure 2:**
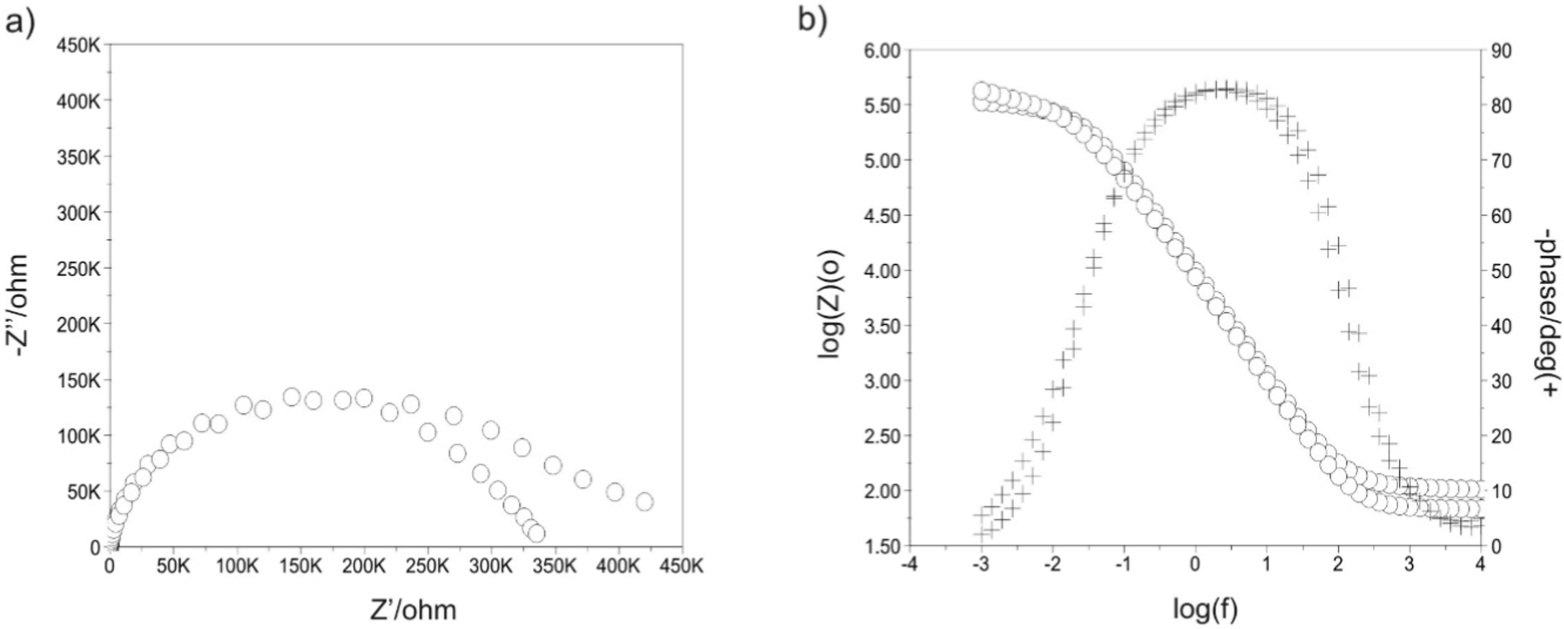
Impedance spectra for the Ti-13Nb-13Zr alloy with TiN layer (a) Nyquist and (b) Bode diagrams. -Impedance spectra illustrating the electrochemical properties of the TiN-coated alloy, showing the influence of the layer in interaction with the PBS solution.

The application of the layer on the Ti-13Nb-13Zr substrate led to reduced electrochemical stability, as evidenced by the lower charge transfer resistance (*R*_ct_). The Ti-13Nb-13Zr specimens with the TiN layer exhibited signs of a porous oxide layer due to interaction with the PBS solution. Notably, a change in the angle of inclination of the linear section in the low-frequency part of the spectrum was observed. Initially, the angle indicated Warburg impedance (45°), which later shifted to an angle typical of membrane-type layers. This change can be attributed to the formation of a biofilm at the interface between the TiN layer and the PBS solution.

Based on this model, two R and CPE elements were connected in series with the solution resistance (*R*_s_) (Figure 3). As a result, partial degradation of the TiN layer in the surface sublayer was seen, while simultaneously membrane-type films were formed on the inner layer (adsorption layer). The passive film and electrical double layer were assumed to exhibit ideal or nonideal capacitive behavior. The pore resistance (*R*_pore_) and the constant phase element of the pore (CPE_pore_) represented the electrical properties of the porous layer, while *R*_ct_ and the constant phase element of the double layer (CPE_dl_) characterized the resistive and nonideal capacitive behavior of the passive film.

**Figure 3:**
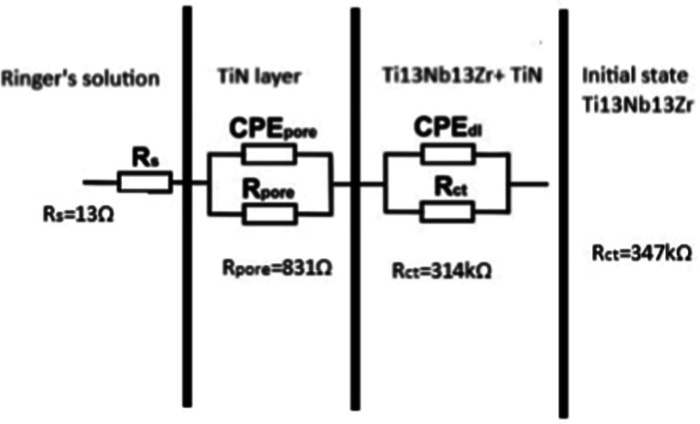
Electrical equivalent circuits for the corrosion system consisting of Ti-13Nb-13Zr alloy in PBS solution for Ti-13Nb-13Zr + TiN. An equivalent electrical circuit model describing the corrosion mechanism at the TiN layer-PBS interface during EIS analysis.

The best fit of the model spectra to the experimentally determined impedance spectra in the PBS solution for the specimens with TiN layers was achieved using a simple equivalent circuit with one time constant comprising four electrical elements (Figure 3).

In this circuit, *R*_s_ represents the resistance of the electrolyte (PBS solution), *R*_ct_ denotes the resistance to electric charge transfer at the interface between the TiN layer and the PBS solution, and CPE_dl_ describes the electrical properties of the double layer at this interface. This auxiliary electric system (Figure 3) was employed to analyze the experimentally obtained impedance spectra for Ti-13Nb-13Zr and Ti-13Nb-13Zr with TiN layers created by using PVD.

### 3.3 Scratch Test

Subsequently, scratch tests were performed, and variations in critical force (as shown in Figure 4 and Table 6), which indicated adhesion, were recorded. The average force required to damage the layer after the complete failure of the Ti-13Nb-13Zr specimen with the TiN layer (Ti-13Nb-13Zr + TiN) was noted, with a chip load (Lc_2_) of 7.64(86) and with a critical load (Lc_3_) of 16.95(31) N. In contrast, chipping (Lc_2_) was observed for Ti-13Nb-13Zr + TiN.

**Figure 4:**
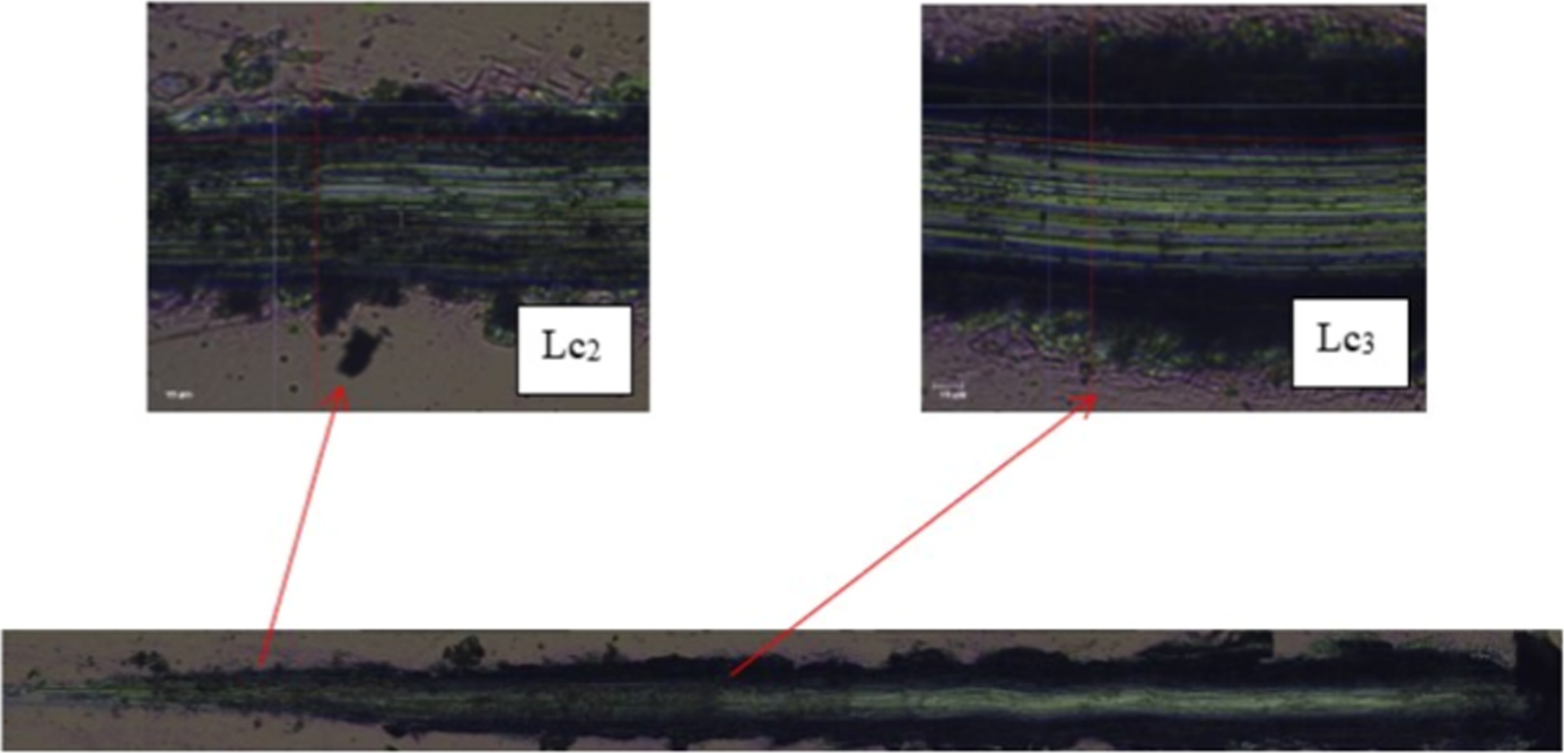
Optical micrographs of the adhesion tests for a Ti-13Nb-13Zr specimen with the TiN layer. Optical micrographs showing layer damage and chipping on the TiN-coated specimen during the scratch adhesion test.

**Table 6:** Results Adhesion Tests for a Ti13Nb13Zr Sample with the TiN Layer

method of specimen surface preparation	layer damage	average force F [N]
Ti-13Nb-13Zr + TiN	chip of Lc_2_	7.64(86)
	complete destruction of Lc_3_	16.95(31)

Complete failure of the layer was observed in the Ti-13Nb-13Zr + TiN specimen. Furthermore, the absence of an acoustic emission (AE) signal in the specimens suggested that the bonding energy between the layer and the substrate was insufficient. Additionally, SEM observations conducted after the scratch test confirmed the complete destruction (Lc_3_) of the layer (Figure 5).

**Figure 5:**
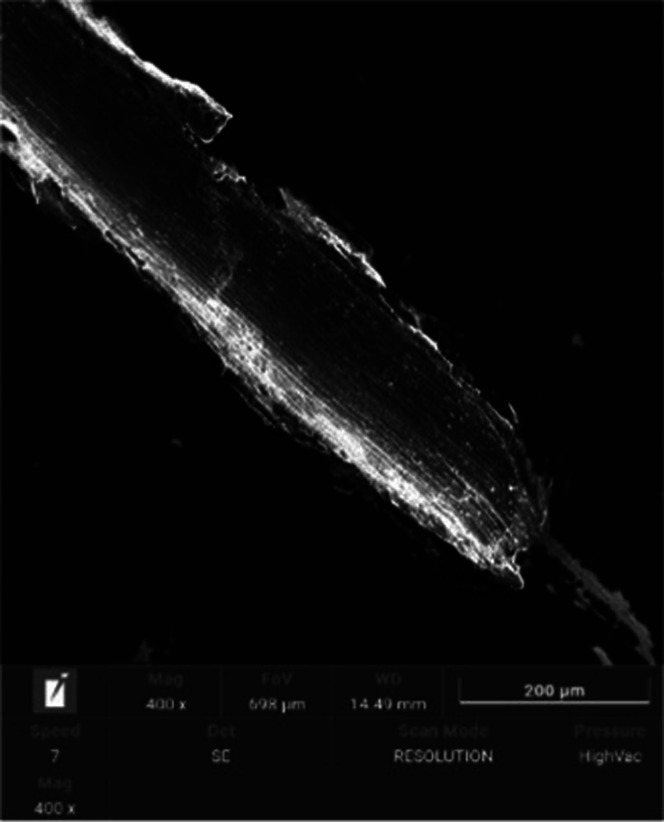
SEM micrograph of the Ti-13Nb-13Zr + TiN specimen. Surface after the scratch test (SEM area taken at 400×). SEM image displaying the surface damage and failure mode of the TiN layer after the scratch test.

### 3.4 Tribology

To assess the tribological properties of the materials, friction tests were carried out with results presented in Figures 6 and 7. The average abrasion coefficients for the tested specimens are presented in Figure 7, derived from tribological tests conducted with an Al_2_O_3_ ball. For the specimens coated with TiN, there was a reduction in the coefficient of friction and an enhancement in the abrasion resistance. The application of the layer resulted in a notable decrease in the friction coefficient for the Ti-13Nb-13Zr + TiN specimens compared to that for the initial state Ti-13Nb-13Zr specimens. In this case, the friction coefficient (μ) of the test specimens was influenced by the materials (surface layer) used. Tribological testing methodology is closely related to the use of the given alloy, thus the specimen within the skeletal system, while simulating implant-bone contact in the case of, for example, a ball-and-socket joint. Reducing the coefficient of friction by applying a TiN layer leads to better results in the context of use as a bone implant, which can be of great importance for surgery.

**Figure 6:**
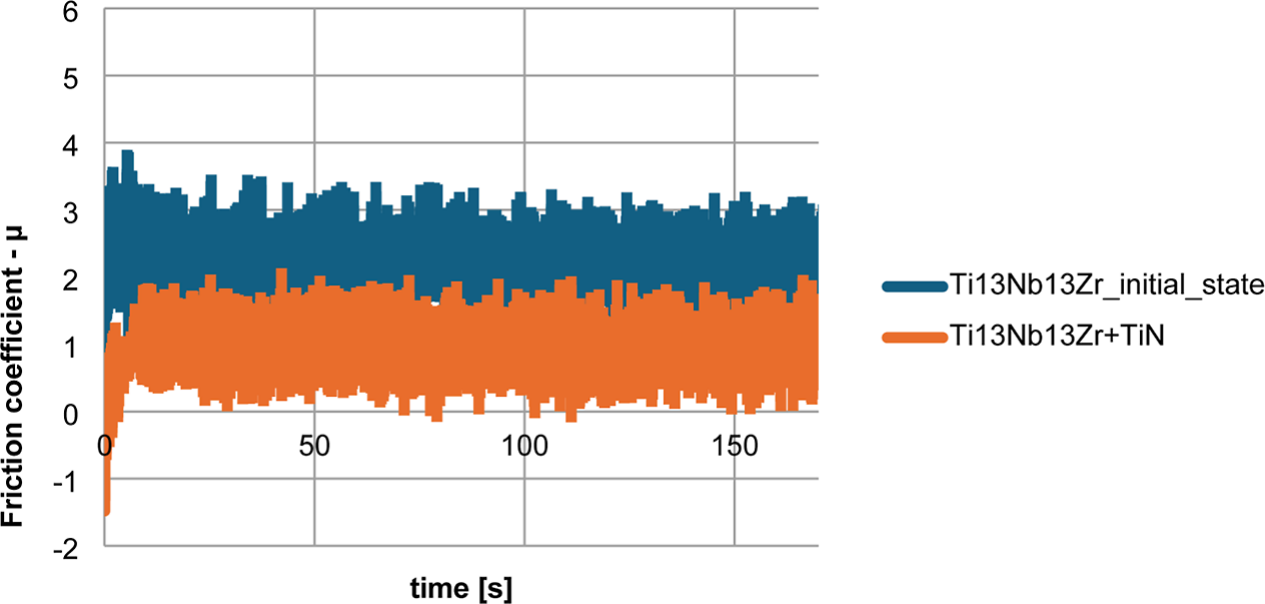
Tribological test results. Friction test results comparing the behavior of uncoated and TiN-coated specimens under wear conditions.

**Figure 7:**
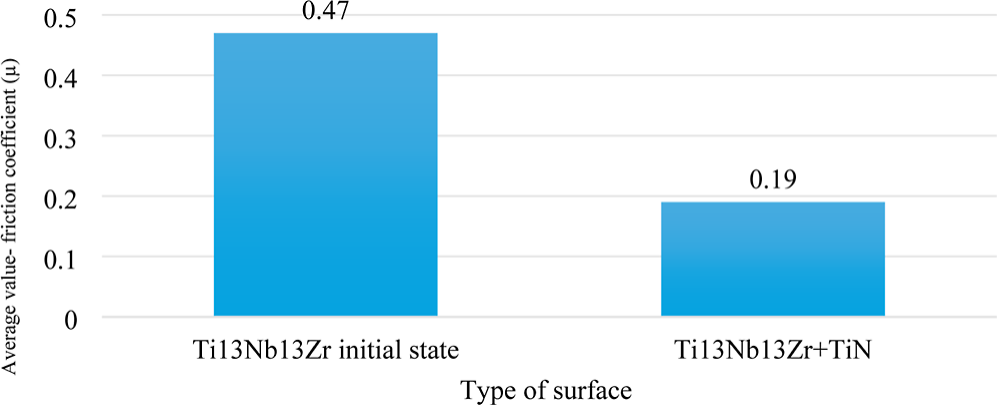
Average coefficient of friction, μ, values for the Ti-13Nb-13Zr alloy and Ti-13Nb-13Zr TiN specimens. Comparison of average friction coefficients, indicating improved wear resistance due to the TiN layer.

### 3.5 SEM Observations—Surface before and after Coating

To assess the morphological differences between the uncoated and TiN-coated surfaces before tribological testing, SEM observations were performed as shown in Figures 8 and 9. The uncoated Ti-13Nb-13Zr surface exhibited a relatively smooth topography with visible grinding marks, while the TiN-coated surface demonstrated a more homogeneous and compact morphology due to the presence of the TiN layer. These images confirm the successful deposition of the TiN layer and its influence on surface characteristics.

**Figure 8:**
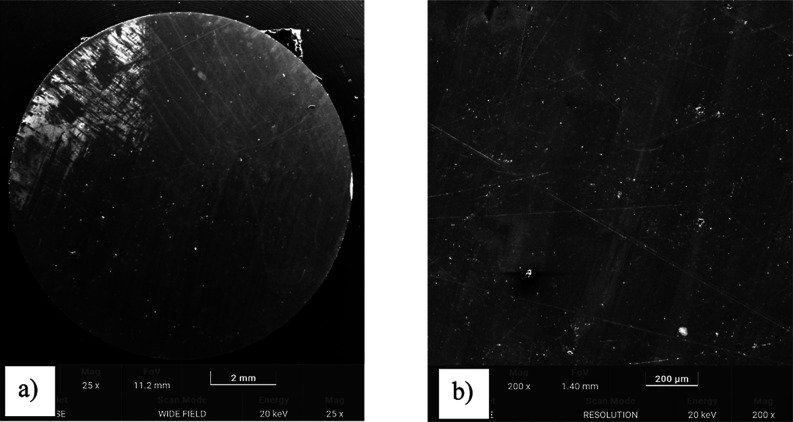
SEM images of the Ti-13Nb-13Zr alloy before TiN coating (magnification: (a) 25×, (b) 200×).

**Figure 9:**
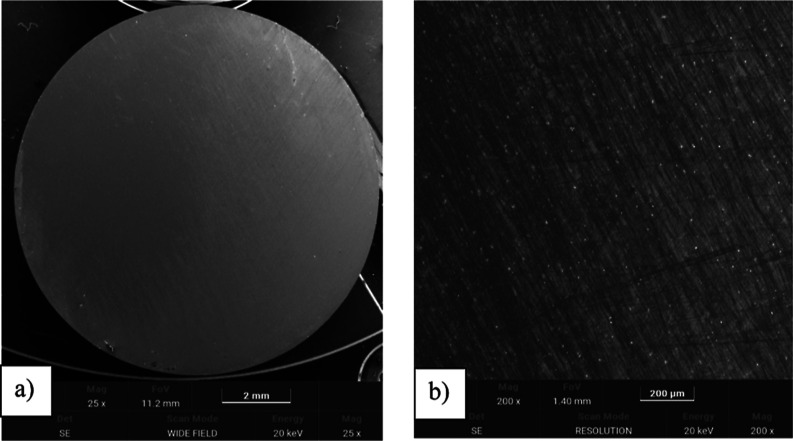
SEM images of the Ti-13Nb-13Zr alloy after TiN coating (magnification: (a) 25× and (b) 200×. Smooth surface morphology with visible polishing traces before surface modification.

Additionally, SEM observations conducted after tribological testing revealed abrasion marks (Figures 10 and 11).

**Figure 10:**
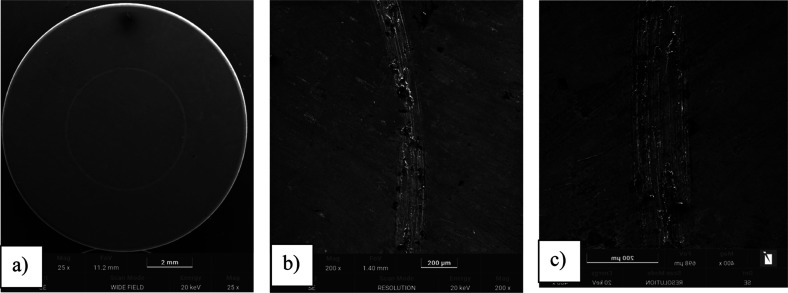
The surface of the Ti-13Nb-13Zr alloy (initial state) after the friction pair abrasion test, with SEM magnifications of (a) 25×, (b) 200×, and (c) 400×. SEM images of the uncoated sample after the abrasion test, showing wear tracks at different magnifications.

**Figure 11:**
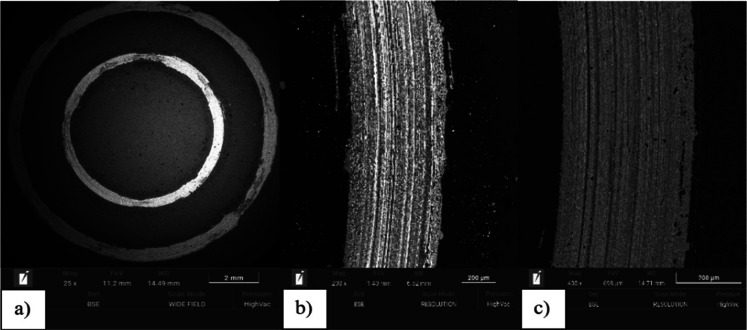
The surface of the Ti-13Nb-13Zr alloy with the TiN layer after the friction pair abrasion test, with SEM magnifications of (a) 25×, (b) 200×, and (c) 400×. SEM images of the TiN-coated surface after the abrasion test, highlighting improved wear protection at multiple magnifications.

### 3.6 Wettability

The application of the layer resulted in changes in the surface characteristics and the contact angle as shown in Figure 12. The average contact angle values for the tested specimens using distilled water are shown in Figure 10.

**Figure 12:**
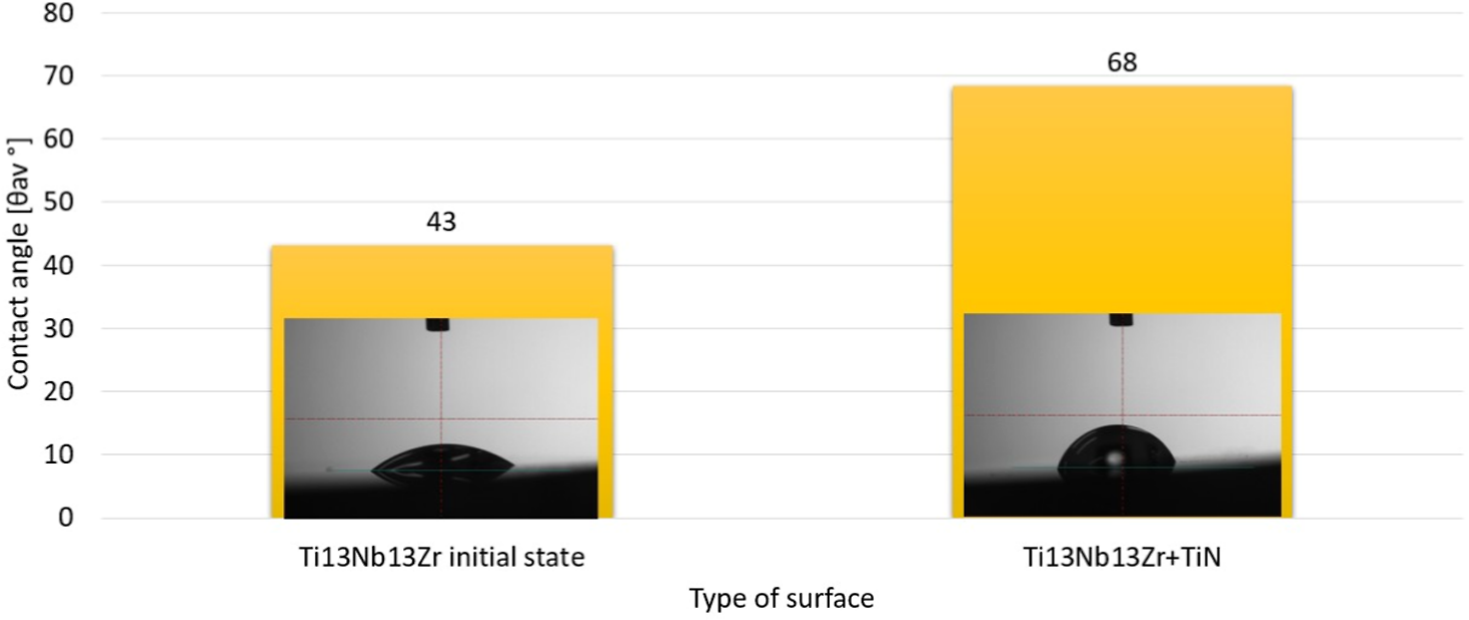
Contact angle images were taken during the wettability measurements for the (a) Ti-13Nb-13Zr initial state and (b) Ti-13Nb-13Zr TiN. Contact angle measurements demonstrating changes in surface wettability after TiN coating, relevant to cell adhesion and biocompatibility.

The observed differences in contact angle values are influenced by the various properties of the liquid used in the tests. A contact angle of 90° distinguishes biomaterials as either hydrophilic or hydrophobic, which affects cell adhesion and the behavior of droplets during testing (Figure 12). For the untreated Ti-13Nb-13Zr specimen, the wetting angle remained unchanged regardless of the surface characteristics, while the specimens with layers retained a hydrophilic nature, with contact angles between 0° and 90°. The Ti-13Nb-13Zr + TiN specimen had an increased contact angle of 68° rather than the initial angle of 43° (Figure 12). The fact that the specimens kept hydrophilic surfaces is closely linked to their potential for cell proliferation and osseointegration.

To improve biomaterials used for bone implants, which should be characterized by good corrosion resistance, appropriate mechanical and electrical properties, high metallurgical quality, surface uniformity, biocompatibility, abrasion resistance, and relatively low production costs, we modified their surfaces with PVD. Electrical properties, such as high electrochemical potential and good electrical conductivity are key in titanium implants. These properties affect the process of biocorrosion and osseointegration or incorporation of the implant into the bone. This process also enhances the antibacterial properties of the Ti-13Nb-13Zr alloy and mitigates the negative effects of the bacterial biofilm formation. Specific surface treatment conditions were established to achieve the desired physicochemical characteristics of the Ti-13Nb-13Zr surface. The preparation of the specimens included grinding with varying grades of sandpaper, followed by polishing. The next step was the deposition of TiN using PVD. Argon gas was utilized to generate plasma for the deposition process, introduced at a power level of 25%. The pitting corrosion resistance and EIS were assessed using a potentiodynamic approach, leading to the generation of polarization curves for analysis. The TiN layer applied via PVD gave no improvement but neither was a deterioration in the electrochemical properties of the Ti-13Nb-13Zr alloy noted. Mechanical property evaluations of the surface-modified Ti-13Nb-13Zr alloy included adhesion and abrasion resistance tests. The results indicated that the layers showed enhanced adhesion when subjected to the PVD process. Piotrowska and Madej^13^ conducted similar examination based on microscopic observations and also recorded changes in friction coefficient (μ) and acoustic emission (AE), the deposited layer was found to have high adhesion to the substrate.

The scratch test of the TiN/Ag coating showed that the coating did not delaminate as a result of the applied load, as in our examination. There were no characteristic jumps in the coefficient of friction curves to indicate chipping or peeling of the coating in any of the cases examined. In turn, the acoustic emission diagram showed that at a load of approximately 14.5 N, the first cracks in the coating began to appear. In comparison, in our examination, the first cracks showed with using a load 2 times lower. Also tribological tests demonstrate the lubricant’s positive effect on both friction pairs. The motional resistance recorded for the Ti13Nb13Zr titanium alloy was approximately TiN/Ag coating and 70% lower than the values obtained with technical dry friction. In comparison Ti13Nb13Zr with TiN coating was ensured about 50% lower coefficient friction than without the coating using.

Scratch tests recorded changes in acoustic emissions, revealing localized delamination rather than complete failure. Abrasion resistance tests highlighted the significant positive impact of the TiN layer on the Ti-13Nb-13Zr alloy, which meant that the coefficient of friction against an Al_2_O_3_ ball was about two times lower than that of the uncoated alloy. The adhesion strength of TiN coating was examined by Shah and co-workers^14^ by means of scratch tests and the coating failed at the critical load range of 24.4–28.5 N. It is higher than our values, but it is a connection between different temperatures of conducting the process of PVD. It was also observed that the friction of coefficient graphs for all conditions began at a certain value between 0.25 and 0.45 and its higher coefficient than ours, so it can be claimed that in this case we have better adhesion of coating. The authors were explained that nonzero initial coefficient of friction values may be attributed to the resistance force faced by the indenter tip to overcome the roughness of the thin coating. Tillmann^11^ et al. reported that using a TiN layer also decreased the coefficient of friction, giving an improvement in wear resistance. The final phase of the study assessed the surface wettability. All the tested variants exhibited hydrophilic properties, promoting cell adhesion, a crucial factor for orthopedic implants as it influences implant integration into tissue.

## 4 Conclusions

Based on this research, the following conclusions can be drawn:

- Application of the TiN casing did not lead to worse results for Ti-13Nb-13Zr on the pitting device and did not affect other anticorrosion properties.
- Ti-13Nb-13Zr alloy is the appropriate substrate for TiN layer adhesion.
- Application of TiN improved the abrasion resistance of the Ti-13Nb-13Zr alloy, which allows its use within the skeletal system.
- Application of TiN did not affect the surface properties and the Ti-13Nb-13Zr alloy remained hydrophilic, which can affect in future the process of osseointegration and cell proliferation.

Osseointegration is the process through which bone tissue anchors to an implant, while cell proliferation refers to the growth and multiplication of cells. The process of osseointegration, understood as the direct structural and functional connection between living bone tissue and the implant surface, plays a key role in the stability and long-term functionality of implants. Our results suggest that studied parameters such as abrasion resistance, scratch test, and wettability in relation to implant surface modification, among others, may promote faster onset of osseointegration, potentially reducing healing time and increasing implant effectiveness. Moreover, the nondeterioration of corrosion resistance and other potentiodynamic properties, confirms the performance of the coating-as-neutral and not altering these properties, which is important in the context of long-term implants.

Further continuation of current work and research into cytotoxicity testing, biological testing, or cell proliferation may lead to solutions to problems and such improvements would lead to improved efficacy, lower complication rates, and better quality of life for patients.
